# Comparisons of Musculoskeletal Disorders among Ten Different Medical Professions in Taiwan: A Nationwide, Population-Based Study

**DOI:** 10.1371/journal.pone.0123750

**Published:** 2015-04-10

**Authors:** Shu Yi Wang, Liang Chun Liu, Ming Chi Lu, Malcolm Koo

**Affiliations:** 1 Department of Rehabilitation, Dalin Tzu Chi Hospital, Buddhist Tzu Chi Medical Foundation, Chiayi, Taiwan; 2 Division of Allergy, Immunology and Rheumatology, Dalin Tzu Chi Hospital, Buddhist Tzu Chi Medical Foundation, Chiayi, Taiwan; 3 School of Medicine, Tzu Chi University, Hualien, Taiwan; 4 Department of Medical Research, Dalin Tzu Chi Hospital, Buddhist Tzu Chi Medical Foundation, Chiayi, Taiwan; 5 Dalla Lana School of Public Health, University of Toronto, Ontario, Canada; Örebro University, SWEDEN

## Abstract

**Objective:**

Medical personnel are at risk of musculoskeletal disorders but little is known whether the risk of musculoskeletal disorders were different among various medical professions. Therefore, this study compared the risk of musculoskeletal disorders among personnel of 10 different medical professions in Taiwan using a nationwide health claims database.

**Methods:**

Data from the 2000–2010 Taiwan National Health Insurance Research Database were used to identify personnel of 10 different medical professions. Diagnoses based on the International Classification of Diseases, Ninth Revision, Clinical Modification (ICD-9-CM) were used to identify eight different musculoskeletal disorders that occurred after the license issuance date. Cox proportional hazards model was used to compare the risk of eight musculoskeletal disorders among the 10 different medical professions using dentists as the reference category.

**Results:**

A total of 7,820 medical personnel were included in the analysis. Using dentists as the reference category, physical therapists showed a significantly higher risk of all eight musculoskeletal disorders (ranging from 1.59 [*p* = 0.032] in sprains and strains of other and unspecified parts of back to 2.93 [*p* < 0.001] in spondylosis and allied disorders).

**Conclusions:**

Compared with dentists, a profession that already known to suffer from high rates of work-related musculoskeletal disorders, physical therapists, registered nurses, and doctors of Chinese medicine showed an even higher risk of musculoskeletal disorders.

## Background

The risk of work-related musculoskeletal disorders is high among various healthcare professionals [1–4]. Musculoskeletal disorders can lead to increased health care use [5], reduced work productivity, and lower levels of health-related quality of life [6]. Previous studies indicated that prevalence of musculoskeletal complaints, particularly low back and neck-shoulder, were high in nurses and X-ray technologists [7,8]. A study conducted on 1,600 employees in six hospitals in Turkey reported that nurses had the highest prevalence of low back pain. Age, female sex, smoking, occupation, perceived work stress, and heavy lifting were significant and independent risk factors for low back pain [1]. Another cross-sectional study conducted on dentists, laboratory technicians, nurses, physicians, and physiotherapists in a tertiary care hospital in India revealed that working in the same position for long periods, working in awkward positions, and handling a large number of patients were commonly reported risk factors for work-related musculoskeletal disorders [9].

High prevalence for back pain, neck, shoulder, and hand-wrist region complaints among dental professionals had also been reported [10,11]. Results from a mail survey of 3,297 randomly selected physical and occupational therapists living in Wisconsin reported that their annual incidence rate of work-related injuries were comparable to that among workers employed in heavy manufacturing [12]. A systematic review of 65 studies revealed that a high prevalence of upper limb musculoskeletal disorders in dental professionals, nurses, and laboratory technicians but not in physiotherapists [13].

Although a number of studies have previously examined the risk of musculoskeletal disorders among healthcare workers, many of them used a cross-sectional study design and were based on self-reporting and recall of injuries. The information collected is prone to recall bias. Therefore, this study used a cohort study design to investigate the risk of musculoskeletal disorders among medical personnel, based on the data available from a nationwide health claims database in Taiwan. In addition, by comparing the types of musculoskeletal disorders among 10 different medical professions, the results could be used for targeting relevant ergonomics improvements and interventions to specific groups of medical personnel.

## Methods

### Data source

The Taiwan National Health Insurance Research Database (NHIRD) contains claim data for reimbursement from the National Health Insurance (NHI) program, which is a mandatory social health insurance system implemented in Taiwan beginning in 1995 [14]. The NHI is a social insurance system funded by the premiums paid by the insured, employers, and the government. The insured are classified into different categories to determine their premiums. Insured individuals who are fully employed, pay premiums based on their categories and their salary. Employers and the self-employed pay 100% of their premiums. The government subsidizes 100% of the premiums for low-income households, veterans and inmates in correctional facilities [15]. Since NHI is a mandatory single-payer program, the NHIRD comprehensively includes claim data on both outpatient and inpatient services for nearly the entire population of Taiwan.

As of the end of 2011, 23.20 million out of 23.22 million (99.9%) residents in Taiwan were enrolled in the system [16,17]. As of 2010, 92.1% of all health care facilities in Taiwan were contracted by the NHI system [15].

Under the NHI program, the co-payments for a single ambulatory visit range from only US$1.7 at local clinics to US$15 at medical centers. Other costs including ambulatory medical care such as physician consultation fees, visits to emergency departments, medical procedures, pharmacy expenditure, laboratory diagnosis tests, imaging examination, and rehabilitation therapy are all covered by the NHI. The full dataset of NHIRD contains all ambulatory claims, inpatient claims, details of ambulatory care, inpatient orders, and prescriptions dispensed at contracted pharmacies. The National Health Insurance Administration routinely performs cross-checks and validations of medical claims using a fully automated procedural review track and a professional peer review track to ensure the accuracy of the NHIRD diagnostic coding [15].

In this study, we used the Longitudinal Health Insurance Database 2000 (LHID2000), one of the NHIRD dataset to obtain information on the diagnoses of ambulatory medical visits that occurred during January 1, 2000 to December 31, 2010.

Specifically, we used the "Ambulatory Care Expenditures by Visits" (official abbreviation: CD) datafile to obtain diagnosis for outpatient visits. The LHID2000 contains the claim of one million beneficiaries randomly sampled from approximately 23.7 million beneficiaries of the NHIRD in the year 2000. There were no significant differences in the distributions of age, sex, or insured amount between the individuals in the LHID2000 and its source population [14].

We also used the "Registry for Medical Personnel" (official abbreviation: PER) datafile to identify individuals of the following 10 health professions: dentists, doctors of Chinese medicine, dentists, medical radiation technologists, medical technologists, occupational therapists, pharmacist, physical therapists, physicians, registered professional nurse, and registered nurses. We used the data field "work_status" in the PER datafile to exclude individuals whose working status were recorded as inactive and we also excluded those with musculoskeletal disorders before the date when they obtained their medical licenses. In addition, we included only those individuals who obtained their licenses between the ages of 20 to 39 years.

Since the NHIRD files contain only de-identified secondary data, the need for informed consent from individual subjects was waived. The study protocol was reviewed and approved by the institutional review board of the Dalin Tzu Chi Hospital, Buddhist Tzu Chi Medical Foundation, Taiwan (No. B10202020).

### Study design

A cohort study design was used to follow the eligible medical personnel from the date of their medical license issuance, which is a data field available in the PER datafile. They were followed-up until the date of diagnosis of musculoskeletal disorders or the last date of their ambulatory medical visits in the LHID2000, whichever came first.

### Outcome measures

The musculoskeletal disorders outcome measures were identified based on the International Classification of Diseases, Ninth Revision, Clinical Modification (ICD-9-CM) code available in the "ACODE_ICD9_1", "ACODE_ICD9_2", and "ACODE_ICD9_3" data fields in the CD datafile of the LHID2000. The eight musculoskeletal disorders were spondylosis and allied disorders (ICD-9-CM 721.x), intervertebral disc disorders (ICD-9-CM 722.x), other disorders of cervical region (ICD-9-CM 723.x), other and unspecified disorders of back (ICD-9-CM 724.x), sprains and strains of shoulder and upper arm (ICD-9-CM 840.x), sprains and strains of wrist and hand (ICD-9-CM 842.x), sprains and strains of knee and leg (ICD-9-CM 844.x), and sprains and strains of other and unspecified parts of back (ICD-9-CM 847.x).

### Statistical analysis

Summary statistics are expressed as frequencies and percentages for categorical data or means and standard deviations for continuous variables, as appropriate. The hazard ratios for musculoskeletal disorders were estimated by Cox proportional hazards models [18], adjusting for sex and age at medical license issuance. The time variable used in the Cox proportional hazards models was the interval between license date and the date of diagnosis of musculoskeletal disorders, or the last date of their ambulatory medical visits for those without musculoskeletal disorders. The adjusted hazard ratios of musculoskeletal disorders among the different medical professions were compared using dentists as the reference group. Dentists were chosen as the reference group for two reasons. First, information on occupation is not available in the NHIRD except in cases where the insurers are licensed medical personnel. The lifestyle, knowledge of disease prevention, and workload of medical personnel are assumed to be more homogeneous among different medical professions compared with non-medical jobs. Second, dentists are known to have a high rate of work-related musculoskeletal disorders and by using them as the reference group, it allows the evaluation of whether the other nine medical professions have a higher risk beyond that of the dental profession.

The proportional hazards assumption was evaluated by including an interaction term between time and the medical profession variable in the Cox regression model. A two-tailed *p* value of < 0.05 was considered statistically significant. All statistical analyses were conducted using IBM SPSS Statistics software package, version 21.0 (IBM Corp., Armonk, NY, USA).

## Results

A total of 7,820 medical personnel were identified and included in the analysis. The mean age at license issuance was 26.6 years (standard deviation 5.1 years) and 80.7% were females (Table 1). The adjusted hazard ratios (AHR) of the eight musculoskeletal conditions among the medical personnel are showed in Table 2. The dentist group is listed as the reference group on the left followed by the other nine groups of medical personnel arranged alphabetically across the table. Compared with dentists, physical therapists had elevated risks in all eight musculoskeletal disorders, with AHR ranging from 1.59 (*p* = 0.032) in sprains and strains of other and unspecified parts of back (ICD-9-CM 847.x) to 2.93 (*p* < 0.001) in spondylosis and allied disorders (ICD-9-CM 721.x). Registered nurses also had significantly higher risks in five musculoskeletal disorders, with AHR ranging from 1.52 (*p* = 0.022) in sprains and strains of other and unspecified parts of back (ICD-9-CM 847.x) to 2.22 (*p* < 0.001) in other and unspecified disorders of back (ICD-9-CM 724.x). Doctors of Chinese medicine had a higher risk in sprains and strains but significantly lower risk in intervertebral disc disorders (AHR = 0.13, *p* = 0.006) and marginally lower risk in spondylosis and allied disorders (AHR = 0.34, *p* = 0.051) compared with dentists. In addition, medical radiation technologists had risks of musculoskeletal disorders similar to that of dentists. Physicians also had similar risks of musculoskeletal disorders compared with dentists except that they had a significantly lower risk of sprains and strains of other and unspecified parts of back (ICD-9-CM 847.x) (AHR = 0.47, *p* < 0.001).

**Table 1:** Demographic data of study subjects.

Category of medical personnel	Number	Licensed age (yrs), Mean (SD)	Sex, Number (%)	
			Male	Female
**All medical personnel**	7820	26.6 (5.1)	1508 (19.3)	6312 (80.7)
				
Doctor of Chinese medicine	118	33.3 (3.7)	81 (68.6)	37 (31.4)
Dentist	231	28.8 (4.7)	148 (64.1)	83 (35.9)
Medical radiation technologist	198	26.4 (4.5)	83 (41.9)	115 (58.1)
Medical technologist	281	27.0 (4.6)	75 (26.7)	206 (73.3)
Occupational therapist	114	25.5 (3.2)	44 (38.6)	70 (61.4)
Pharmacist	578	28.2 (5.1)	198 (34.3)	380 (65.7)
Physical therapist	195	26.2 (3.9)	94 (48.2)	101 (51.8)
Physician	918	30.2 (4.4)	711 (77.5)	207 (22.5)
Registered professional nurse	3613		58 (1.6)	3555 (98.4)
Registered nurse	1574	25.6 (4.9)	16 (1.0)	1558 (99.0)

SD: standard deviation.

**Table 2:** Risks of musculoskeletal disorders among medical professions in Taiwan.

**Condition (ICD-9-CM code)**	**Category of medical personnel**									
	Dentist	Doctor of Chinese medicine	Medical radiation technologist	Medical technologist	Occupational therapist	Pharmacist	Physical therapist	Physician	Registered professional nurse	Registered nurse
**Spondylosis and allied disorders** (721.x)										
AHR (95% CI)	1.00	0.34 (0.11–1.01)	1.30 (0.63–2.67)	1.72 (0.89–3.30)	0.88 (0.29–2.65)	1.33 (0.73–2.41)	2.93 (1.55–5.52)	1.04 (0.60–1.81)	1.75(1.00–3.08)	1.81 (1.01–3.24)
*p* value	-	0.051	0.480	0.106	0.814	0.354	<0.001	0.900	0.052	0.046
**Intervertebral disc disorders** (722.x)										
AHR (95% CI)	1.00	0.13 (0.03–0.56)	1.50 (0.85–2.65)	0.89 (0.49–3.98)	2.61 (1.40–4.83)	1.02 (0.61–1.70)	2.63 (1.56–4.43)	0.97 (0.61–1.54)	1.38 (0.86–2.20)	1.39 (0.86–2.26)
*p* value	-	0.006	0.163	0.726	0.002	0.943	<0.001	0.889	0.182	0.178
**Other disorders of cervical region** (723.x)										
AHR (95% CI)	1.00	1.86 (0.98–3.14)	0.77 (0.70–2.62)	1.22 (1.25–3.64)	0.92 (0.68–3.84)	1.37 (1.56–3.70)	1.91 (1.99–5.62)	0.57 (1.87–5.70)	1.14 (1.33–3.01)	1.52 (1.74–4.05)
*p* value	-	0.072	0.504	0.552	0.865	0.276	0.046	0.059	0.626	0.138
**Other and unspecified disorders of back** (724.x)										
AHR (95% CI)	1.00	2.35(1.60–3.44)	1.10(0.74–1.64)	1.57 (1.10–2.25)	1.21(0.74–1.98)	1.65(1.20–2.28)	1.67(1.14–2.44)	0.85(0.62–1.17)	1.77 (1.30–2.40)	2.22 (1.63–3.04)
*p* value	-	<0.001	0.638	0.014	0.450	0.002	0.008	0.315	<0.001	<0.001
**Sprains and strains of shoulder and upper arm** (840.x)										
AHR (95% CI)	1.00	4.04(1.91–8.57)	1.65(1.04–3.29)	2.32(0.64–2.19)	1.45(1.08–4.43)	1.64(1.24–2.79)	2.79(1.55–4.51)	0.86(3.51–9.41)	1.52(1.24–2.79)	2.16(1.60–3.70)
*p* value	-	<0.001	0.223	0.026	0.474	0.170	0.007	0.680	0.215	0.025
**Sprains and strains of wrist and hand** (842.x)										
AHR (95% CI)	1.00	5.16(2.55–10.43)	1.66(0.25–1.69)	1.06(0.71–2.72)	1.96(0.41–3.30)	1.73(0.84–2.42)	2.37(1.66–5.29)	0.90(1.36–5.10)	1.67(0.85–2.20)	2.19(0.97–2.67)
*p* value	-	<0.001	0.187	0.885	0.126	0.103	0.020	0.750	0.111	0.016
**Sprains and strains of knee and le**g (844.x)										
AHR (95% CI)	1.00	2.36(0.99–5.62)	0.58(0.20–1.74)	1.25(0.53–2.95)	1.04(0.32–3.38)	1.27(0.60–2.72)	2.65(1.20–5.89)	0.90(0.43–1.87)	1.22(0.60–2.49)	1.44(0.70–2.99)
*p* value	-	0.054	0.334	0.618	0.955	0.537	0.016	0.767	0.586	0.327
**Sprains and strains of other and unspecified parts of back** (847.x)										
AHR (95% CI)	1.00	1.79(1.15–2.79)	1.08(0.69–1.69)	1.31(0.87–1.98)	1.39(0.82–2.37)	0.88(0.60–1.29)	1.59(1.04–2.44)	0.47(0.32–0.69)	1.23(0.87–1.75)	1.52(1.06–2.17)
*p* value	-	0.010	0.744	0.202	0.222	0.495	0.032	<0.001	0.244	0.022

ICD-9-CM: International Classification of Diseases, Ninth Revision, Clinical Modification; AHR: adjusted hazards ratio; 95% CI: 95% confidence interval.

## Discussion

High rates of work-related musculoskeletal injury are well documented among medical professionals, and particularly among physical therapists, occupational therapists, dental professionals, and nurses. Through the use of a nationwide, population-based claims dataset, we were able to simultaneously compare the risks of musculoskeletal disorders among 10 groups of medical personnel. The main findings of this study are that, compared with dentists, physical therapists had elevated risks among all eight musculoskeletal disorders and that other and unspecified disorders of back (ICD-9-CM 724.x) was the condition that affected many groups of the medical personnel. It has previously been reported that 82.8% of physical therapists had experienced symptoms of work-related musculoskeletal disorders in at least one body region during the past 12 month and 91% had experienced work-related musculoskeletal pain or discomfort at some time in their working life [3]. Clearly, possession of knowledge on ergonomics alone was insufficient to prevent physical therapists from the potential injuries that are inherent to the nature of their clinical work. Further research is needed to develop preventive strategies specifically for the work settings of physical therapists.

While our findings are generally consistent with previous research that physical therapists [3,19] and nurses [5,20] showed elevated risks of musculoskeletal disorders, an unique aspect of our study is that we showed the risks in these two groups of medical personnel were even higher than those associated with dentists. It is well known that the prevalence of musculoskeletal complaints is high in dentists [2]. Disorders associated with the neck and lower back are regions that commonly require medical treatment [21]. It was anticipated that certain medical professions should show a lower risk of musculoskeletal disorders compared with dentists. However, only sprains and strains of other and unspecified parts of back (ICD-9-CM 847.x) in physicians and intervertebral disc disorders (ICD-9-CM 722.x) in doctors of Chinese medicine showed significantly lower risks compared with dentists. This finding suggests that all these groups of medical personnel had at least similar levels of risks of developing musculoskeletal disorders as dentists. Further studies should investigate why medical professions that did not require regularly lifting and awkward posture are still associated with an increased risk of musculoskeletal disorders.

Findings from this study also show that registered nurses had significantly increased risk of sprains and strains in shoulder and upper arm (ICD-9-CM 840.x), wrist and hand (ICD-9-CM 842.x), knee and leg (ICD-9-CM 844.x), and back (ICD-9-CM 847.x) in addition to spondylosis and allied disorders (ICD-9-CM 721.x) and other unspecified disorders of back (ICD-9-CM 724.x). A longitudinal survey comparing nurses with postal workers and office workers in New Zealand reported that nurses had a substantial prevalence of low back pain, functional-task-disabling knee, wrist/hand pain, and shoulder pain [4]. A survey study on 884 nurses from a teaching hospital in Japan revealed that musculoskeletal disorders were most commonly reported at the shoulder (71.9%), followed by the lower back (71.3%), neck (54.7%), and upper back (33.9%) [22]. An epidemiological study in China on 3,159 nursing personnel found that muscle strain was the most common diagnosis for their low back pain [23]. Previous research has also indicated that low back pain in nurses can be attributed to manual lifting and work stress [24]. In the present study, registered professional nurses had a significant increased risk only in other unspecified disorders of back (ICD-9-CM 724.x) while the risks of the other seven types of musculoskeletal disorders were not significantly different from those of dentists. It is not clear why registered nurses experienced more types of musculoskeletal disorders in comparison with registered professional nurses. Generally, the tasks and workloads between the two are similar within a hospital setting. One possible explanation is that a higher proportion of registered nurses are working in long-term care facilities compared with registered professional nurses. In general, more patient handling activities such as bathing and assisting out of bed are required in long-term care facilities, which are associated with increased risk of musculoskeletal disorders [25]. Another possible explanation is the differences in health care seeking behavior between the two groups. A cross-sectional survey in Taiwan on 80 nurses and 133 nursing aides revealed that nursing aides (7.5%) were significantly less likely to seek medical care for their musculoskeletal disorders compared with nurses (22.5%) [26]. Whether the heath care seeking behavior is different between registered nurses and registered professional nurses in Taiwan will require further studies.

Doctors of Chinese medicine showed significant decreased risk of intervertebral disc disorders (ICD-9-CM 722.x) (AHR = 0.13, *p* = 0.006) and marginally decreased risk of spondylosis and allied disorders (AHR = 0.34, *p* = 0.051) compared with dentists. This finding is consistent with previous research indicating that doctors of Chinese medicine had significantly reduced risks of various morbidities compared with the general population. A study using the 1998–2002 data from NHIRD found that the risk of all causes morbidities was significantly lower in doctors of Chinese medicine compared with the general population (odds ratio [OR] = 0.36, 95% confidence interval [CI] = 0.33–0.40). In addition, the risk of musculoskeletal disorders (ICD-9-CM 710.x to 739.x) was also significantly lower in the former group (OR = 0.40, 95% CI = 0.38–0.42). The authors suggested that a higher socioeconomic status and self-care conduct among doctors of Chinese medicine may explained the reduced in morbidity risk [27]. Nevertheless, our study also revealed a significant increased risk of other unspecified disorders of back (ICD-9-CM 724.x) and increased risk of sprains and strains among doctors of Chinese medicine. One possible explanation for this finding is that certain therapeutic procedures of Chinese medicine that involve manipulative techniques and acupuncture may increase the risk of sprains and strains.

Several limitations should be taken into account when interpreting the results from this study. First, since the data source is based on a claim database, any musculoskeletal conditions that were treated with self medication are not known. A cross-sectional study of 1,509 nurses from two public hospitals in Rio de Janeiro reported that self-medication was used by 7.7% of the nurses in the previous week for their musculoskeletal-related conditions [28]. Second, the diagnoses were based solely on ICD-9-CM codes. Nevertheless, the Taiwan Bureau of National Health Insurance routinely audits random samples of medical claims to ensure their accuracy. Third, due to the lack of detailed clinical data in the NHIRD, no information regarding the causes of musculoskeletal disorders is available. The possible role of work-related stress or other personal factors cannot be evaluated.

## Conclusions

The present study is, to our knowledge, the first to compare the risk of musculoskeletal disorders among medical personnel of different health professions using a population-based dataset. Overall, our findings indicating that physical therapists, nurses, and doctors of Chinese medicine were at increased risks of various musculoskeletal disorders above those experienced by dentists. Ergonomics improvements and interventions that target to specific groups of medical personnel may be required to reduce their risk of musculoskeletal disorders.

## References

[1] Karahan A, Kav S, Abbasoglu A, Dogan N. Low back pain: prevalence and associated risk factors among hospital staff. J Adv Nurs. 2009;65: 516–524. 10.1111/j.1365-2648.2008.04905.x 19222649

[2] Alexopoulos EC, Stathi IC, Charizani F. Prevalence of musculoskeletal disorders in dentists. BMC Musculoskel Dis. 2004;5: 16. 10.1186/1471-2474-5-16 PMC441388 15189564

[3] Cromie JE, Robertson VJ, Best MO. Work-related musculoskeletal disorders in physical therapists: prevalence, severity, risks, and responses. Phys Ther. 2000;80: 336–351. 10758519 10.1093/ptj/80.4.336

[4] Harcombe H, Herbison GP, McBride D, Derrett S. Musculoskeletal disorders among nurses compared with two other occupational groups. Occup Med. 2014;64: 601–607. 10.1093/occmed/kqu117 25149117

[5] IJzelenberg W, Burdorf A. Risk factors for musculoskeletal symptoms and ensuing health care use and sick leave. Spine. 2005;30: 1550–1556. 15990672 10.1097/01.brs.0000167533.83154.28

[6] McDonald M, DiBonaventura Md, Ullman S. Musculoskeletal pain in the workforce: the effects of back, arthritis, and fibromyalgia pain on quality of life and work productivity. J Occup Environ Med. 2011;53:765–770. 10.1097/JOM.0b013e318222af81 21685799

[7] Bos E, Krol B, van der Star L, Groothoff J. Risk factors and musculoskeletal complaints in non-specialized nurses, IC nurses, operation room nurses, and X-ray technologists. Int Arch Occup Environ Health. 2007;80: 198–206. 16799823 10.1007/s00420-006-0121-8

[8] Yassi A, Lockhart K. Work-relatedness of low back pain in nursing personnel: a systematic review. Int J Occup Environ Health. 2013;19: 223–244. 10.1179/2049396713Y.0000000027 23885775

[9] Yasobant S, Rajkumar P. Work-related musculoskeletal disorders among health care professionals: A cross-sectional assessment of risk factors in a tertiary hospital, India. Indian J Occup Environ Med. 2014;18:75–81. 10.4103/0019-5278.146896 25568602 PMC4280781

[10] Morse T, Bruneau H, Dussetschleger J. Musculoskeletal disorders of the neck and shoulder in the dental professions. Work. 2010;35: 419–429. 10.3233/WOR-2010-0979 20448321

[11] Hayes M, Cockrell D, Smith DR. A systematic review of musculoskeletal disorders among dental professionals. Int J Dent Hyg. 2009;7: 159–165. 10.1111/j.1601-5037.2009.00395.x 19659711

[12] Darragh AR, Huddleston W, King P. Work-related musculoskeletal injuries and disorders among occupational and physical therapists. Am J Occup Ther. 2009;63: 351–362. 19522144 10.5014/ajot.63.3.351

[13] Occhionero V, Korpinen L, Gobba F. Upper limb musculoskeletal disorders in healthcare personnel. Ergonomics. 2014;57: 1166–1191. 10.1080/00140139.2014.917205 24840049

[14] National Health Research Institute: National Health Insurance Research Database, Taiwan. Available: http://nhird.nhri.org.tw/en/index.htm. Accessed 12 March, 2015.

[15] National Health Insurance Administration: National Health Insurance in Taiwan. 2011 Annual Report. Available: http://www.nhi.gov.tw/resource/Webdata/20774_1_NHIINTAIWAN2011ANNUALREPORT.pdf. Accessed 12 March, 2015.

[16] Directorate General of Budget, Accounting and Statistics, Republic of China: Statistical Yearbook of Republic of China 2011. Insured persons of social insurance. Available: http://eng.stat.gov.tw/public/data/dgbas03/bs2/yearbook_eng/y087.pdf. Accessed 12 March, 2015.

[17] Directorate General of Budget, Accounting and Statistics, Republic of China: Statistical Yearbook of Republic of China 2011. Population by sex, rate of population increase, average persons per household, density and natural increase rate. [http://eng.stat.gov.tw/public/data/dgbas03/bs2/yearbook_eng/y008.pdf. Accessed 12 March, 2015.

[18] Hosmer DW, Lemeshow S. Applied survival analysis. Regression modeling of time to event data New York: Wiley; 1999.

[19] Alperovitch-Najenson D, Treger I, Kalichman L. Physical therapists versus nurses in a rehabilitation hospital: comparing prevalence of work-related musculoskeletal complaints and working conditions. Arch Environ Occup Health. 2014;69: 33–39. 10.1080/19338244.2012.719555 23930794

[20] Chung YC, Hung CT, Li SF, Lee HM, Wang SG, Chang SC, et al Risk of musculoskeletal disorder among Taiwanese nurses cohort: a nationwide population-based study. BMC Musculoskelet Disord. 2013;14: 144 10.1186/1471-2474-14-144 23617330 PMC3637823

[21] Hayes MJ, Smith DR, Taylor JA. Musculoskeletal disorders and symptom severity among Australian dental hygienists. BMC Res Notes. 2013;6: 250 10.1186/1756-0500-6-250 23822098 PMC3704656

[22] Smith DR, Mihashi M, Adachi Y, Koga H, Ishitake T. A detailed analysis of musculoskeletal disorder risk factors among Japanese nurses. J Safety Res. 2006;37: 195–200. 16678854 10.1016/j.jsr.2006.01.004

[23] Chiou WK, Wong MK, Lee YH. Epidemiology of low back pain in Chinese nurses. Int J Nurs Stud. 1994;31: 361–368. 7928124 10.1016/0020-7489(94)90076-0

[24] Yip Y. A study of work stress, patient handling activities and the risk of low back pain among nurses in Hong Kong. J Adv Nurs. 2001;36: 794–804. 11903709 10.1046/j.1365-2648.2001.02037.x

[25] Pompeii LA, Lipscomb HJ, Schoenfisch AL, Dement JM. Musculoskeletal injuries resulting from patient handling tasks among hospital workers. Am J Ind Med. 2009;52: 571–578. 10.1002/ajim.20704 19444808

[26] Feng CK, Chang TH, Chang BW. Patient-handling activities and musculoskeletal disorders among female nursing personnel in long-term care facilities. J Occup Safety Health. 2005;13: 205–214.

[27] Liu SH, Li TH, Lin YL, Shiao YJ, Wu SC, Li CY, et al Lower morbidity and disease risk among the Chinese medicine physicians in Taiwan. Tohoku J Exp Med. 2009;219: 207–214. 19851049 10.1620/tjem.219.207

[28] Barros AR, Griep RH, Rotenberg L. Self-medication among nursing workers from public hospitals. Rev Lat Am Enfermagem. 2009;17: 1015–1022. 20126945 10.1590/s0104-11692009000600014

